# Process Optimization and Modeling of Phenol Adsorption onto Sludge-Based Activated Carbon Intercalated MgAlFe Ternary Layered Double Hydroxide Composite

**DOI:** 10.3390/molecules26144266

**Published:** 2021-07-14

**Authors:** Nuhu Dalhat Mu’azu, Mukarram Zubair, Ihsanullah Ihsanullah

**Affiliations:** 1 Department of Environmental Engineering, College of Engineering, Imam Abdulrahman Bin Faisal University, P.O. Box 1982, Dammam 34212, Saudi Arabia; mzzubair@iau.edu.sa; 2 Center for Environment and Water, Research Institute, King Fahd University of Petroleum and Minerals, Dhahran 31261, Saudi Arabia; engr.ihsan.dir@gmail.com or

**Keywords:** adsorption, ternary layered hydroxides, sewage-based adsorbents, response surface methodology, phenolic wastewater treatment, nanocomposites

## Abstract

A sewage sludge-based activated carbon (SBAC) intercalated MgAlFe ternary layered double hydroxide (SBAC-MgAlFe-LDH) composite was synthesized via the coprecipitation method. The adsorptive performance of the composite for phenol uptake from the aqueous phase was evaluated via the response surface methodology (RSM) modeling technique. The SBAC-MgAlFe-LDH phenol uptake capacity data were well-fitted to reduced RSM cubic model (R^2^ = 0.995, R^2^-adjusted = 0.993, R^2^-predicted = 0.959 and *p*-values < 0.05). The optimum phenol adsorption onto the SBAC-MgAlFe-LDH was achieved at 35 °C, 125 mg/L phenol, and pH 6. Under the optimal phenol uptake conditions, pseudo-first-order and Avrami fractional-order models provided a better representation of the phenol uptake kinetic data, while the equilibrium data models’ fitting follows the order; Liu > Langmuir > Redlich–Peterson > Freundlich > Temkin. The phenol uptake mechanism was endothermic in nature and predominantly via a physisorption process (ΔG° = −5.33 to −5.77 kJ/mol) with the involvement of π–π interactions between the phenol molecules and the functionalities on the SBAC-LDH surface. The maximum uptake capacity (216.76 mg/g) of SBAC-MgAlFe-LDH was much higher than many other SBAC-based adsorbents. The improved uptake capacity of SBAC-LDH was attributed to the effective synergetic influence of SBAC-MgAlFe-LDH, which yielded abundant functionalized surface groups that favored higher aqueous phase uptake of phenol molecules. This study showcases the potential of SBAC-MgAlFe-LDH as an effective adsorbent material for remediation of phenolic wastewater

## 1. Introduction 

The rapid proliferation of petrochemical, pesticides, drugs, polymers, dyes, and many other chemical industries result in an increased discharge of phenol-containing wastewater globally [1]. Phenol is a known hazardous pollutant and a potential carcinogen with a high potential of harming human and other living creatures. This is liable to water quality deterioration while negatively impacting the ecosystem when discharged even at a lower concentration range. This called for setting regulatory discharge thresholds worldwide with the United States Environmental Protection Agency, for instance, setting 0.1 mg/L as the upper limit for phenol in industrial effluents discharges into surface waters [2]. On the other hand, the majority of municipal as well as many industrial treatments employed activated sludge process (ASP) as the primary treatment scheme, resulting in the generation of a huge amount of sludge globally [1,3]. The proper management of sludge is a serious issue that consumes a significant budget of the whole ASP [1]. Thus, a more sustainable strategy needs to be adopted for the management of sewage sludge, with a desirable shift from conventional incineration or landfilling, which are more prone to impact the environment negatively. 

In recent years, conversion of the ASP sludge (ASPS) to activated carbon adsorbents for water pollution control has been identified as an effective approach to sustainable management of the ASPS [1,4,5]. The use of sludge-based activated carbon (SBAC) for the removal of phenols from water has received increased attention for its dual benefits of high phenol uptake with a potential of significant rendering the process [1]. Yet, activated carbon adsorption is still one of the popular and attractive alternative treatment methods associated with the removal of phenol from aqueous phases compared to other treatment methods. This owes to the abundant and diverse classes of activated carbon adsorbents, their lower cost, and process simplicity. However, the recovery hurdles for the spent activated carbon particles from the treated water and of generation of residual by-products are notable demerits of the adsorption process that have driven greater interest in finding new sustainable activated carbons for water pollution control.

Amongst the several emerging high-performance adsorbents for the effective removal of phenol from the aqueous phase, there is the class of special materials known as “layered double hydroxides (LDHs)”. Recently, these materials proved to be exceptional due to their unique characteristics and outstanding performance. LDHs have emerged as effective adsorbents for the aqueous removal of different classes of inorganic and organic contaminants and exhibited higher adsorption capacities compared to many other known adsorbents [6,7]. Their versatility in compositions and higher and adsorptive characteristics in terms of ion exchange, BET surface area, and lower toxicity render them adaptable and attractive materials for potential deployment toward the more efficient removal of pollutants found in water and wastewater. As a consequence, a number of research reports showcase the inherent LDH flexibility and adaptability in decoration and coupling layers of various LDHs layers with a wide range of materials such as bentonite clay [8], polymers (chitosan, starch, etc.) [9], carbonous materials such as carbon nanotube (CNT) biochar, graphene [10], TiO_2_ [11], and date-palm wastes [12], amongst many others [13,14]. This LDH integration was reported to have yielded improved morphology, surface and functional groups, and adsorptive characteristics (active binding sites) of the resulting nanocomposites [7,15,16]. Several recent studies have demonstrated high performances of different LDHs nanocomposites for the aqueous phase uptake of phenols from water and wastewater streams [17,18]. This included NiAl-LDH modified sodium citrate that yielded 95% removal and 77.7 mg/g maximum adsorption capacity for phenol and p-nitrophenol, respectively [19]. Similarly, MgAl was reported to have resulted in better uptake capability for p-nitrophenol (356.4 mg/g) and phenol (82.5 mg/g) [20]. Moreover, Mg-Al-LDH nanocomposites with carbon nanotubes intercalation exhibited excellent potential for the uptake of 4-chlorophenol and phenol from the aqueous phase [21]. 

Recently, Mu’azu et al. [1] reported the utilization of sewage-based activated carbons (SBACs) for the removal of phenolic compounds from the aqueous phase. The utilization of sewage sludge for GAC production for phenol uptake has been reported using physical activation methods that included conventional and microwave heating [22] and CO_2_ [23], yielding maximum adsorption capacities of 32.96–34.36 mg/g and 32.4 mg/g. However, chemical agent activation of SBAC apparently provided high yields with reported capacities of 20.95–81.6 mg/g, 2.01 mmol/g, 17.82–96.15 mg/g, and 26.16 mg/g, respectively when ZnCl_2_ [24,25], citric acid–ZnCl_2_ mixture [26], NaOH [25,27], and H_2_SO_4_ [28] were employed. Recently, Xin et al. [29] employed polymer flocculants from which they obtained a higher phenol uptake of 132.33 mg/g. To improve the capacities for the reported ZnCl_2_ activated SBAC, Muazu et al. [17] synthesized an SBAC binary MgFe-LDH composite from which a higher capacity of 138.69 mg/g down from the earlier report, 20.9 mg/g, was recorded for the SBAC. 

Despite the variety of existing adsorbents for the remediation of a wide range of phenols from the water, the quest for linking novel adsorbents with sustainability in environmental management and water treatment necessitate emerging new research. In this regard, no work so far has reported on the ternary LDH composite with NaOH-based SBAC. Considering that NaOH-based SBAC yielded good comparative capacity for phenol uptake [25], intercalating NaOH-activated SBAC within the layer of a ternary LDH has a high potential for yielding a better SBAC-based adsorbent. 

Response surface methodology (RSM) modeling has become an indispensable tool in scientific evaluative research [30]. As such, it was adopted in this study for the evaluation and optimization under different experimental conditions. Its numerous benefits included the ability to develop response models correlating the operational parameters with their respective interactive, statistical analysis of variances (ANOVA) and estimating the curvature of the response surface while enabling process optimizing of the sorption process with a fewer required number of experimental data points [31,32,33,34].

Thus, this work reports, for the first time, the synthesis of a novel NaOH-based SBAC intercalated MgAlFe ternary-LDH (SBAC-MgAlFe) via a coprecipitation process and evaluation of its adsorptive potentials for the removal of phenol from the aqueous phase. Additionally, the mechanism of phenol uptake onto the SBAC-MgAlFe was studied and elucidated via kinetics as well as equilibrium and regeneration studies. 

## 2. Materials and Method

### 2.1. Sludge and Chemical Reagents

The sludge used was sourced from a tertiary wastewater treatment plant managed by the Saudi Aramco facility located in Dhahran, Saudi Arabia. The collected sample was air dried immediately in an oven for 0.5 days at a temperature of 105 °C. The cooled and dried sample was ground and sieved with a 100 µM mesh size sieve prior to use in the production of the SBAC. The SBAC used in the ternary MgAlFe-LDH composite in this study was produced using NaOH activation from precursor sludge as reported by Musliu et al. [25]. Phenol (C_6_H_6_O), hydrochloric acid (HCl), sodium hydroxide (NaOH), aluminum hexahydrate (Al(NO_3_)_3_·9H_2_O), magnesium (II) nitrate hexahydrate (Mg(NO_3_)_2_·6H_2_O), Iron(III) nitrate nonahydrate (Fe(NO_3_)_3_ Â·9H_2_O), and other chemical reagents obtained from Sigma Aldrich Co (USA) were used as purchased because they are all of high purity (>98.7%). 

### 2.2. Synthesis of Ternary SBAC-MgAlFe LDH Composite

A coprecipitation process as reported by Alagha et al. [35] was employed for the hybridization of the MgAlFe with the NaOH-SBAC to produce the SBAC-MgAlFe-LDH composite. Firstly, for each molar ratio of 0.05:0.05:0.1 of the precursor salts, which were Mg:Al:Fe salts (i.e., 1.3:1.9:4.04 g), 250 mg of the SBAC was homogeneously dispersed in 100 mL of the ionized water–salts mixture via ultrasonication for half an hour. The slurry of the SBAC and the salts mixture was further mixed vigorously with a magnetic stirrer in a reaction flask at a higher temperature of 90 °C for 15 min. Afterwards, the pH was raised and stabilized at 10 ± 0.5 by the gradual dropwise addition of 1M NaOH prior followed by refluxing for 18 h while maintaining the temperature at 90 °C. At the end of the reaction, the produced composite SBAC-MgAlFe was separated from the supernatant water via centrifuge and then washed with deionized water and ethanol and allowed to completely dry in an oven set at 85 °C. 

### 2.3. Characterization of Ternary SBAC-MgAlFe LDH Composite

The morphology and physicochemical characteristics of the SBAC-MgAlFe were determined using a thermogravimetric analyzer instrument (TGA−50 Shimadzu, Tokyo, Japan), Brunauer Emmett Teller (BET, Micromeritics, Tristar II series, Norcross, GA, USA), X-ray diffraction (XRD, D8 advance X-ray instrument, Bruker, Billerica, MA, USA, for 2θ range 70° to 10° and 0.1542 nm wavelength), scanning electron microscopy (SEM, SM−6460LV (Jeol), Tokyo, Japan), Fourier transform infrared (FTIR, Nicolet 6700, Thermo fisher, Waltham, MA, USA, resolution 4 cm^−1^), point of zero charge using the drift method [36], and a HANA, Padova, Italy pH meter.

### 2.4. RSM Experiments Design Matrix and Modeling 

To evaluate the adsorptive performance of the new SBAC-MgAlFe-LDH, RSM was adopted in this study due to its numerous benefits, as mentioned earlier. Under the RSM, a 3^3^ faced centered central composite design (FC-CCD) was adopted for investigating the dependence of maximum adsorption capacity (q_e_) on temperature (A = 25–45 °C), initial phenol concentration (B = 22.4 ± 2.53 to 125 ± 4.52 mg/L), and initial pH (C = 2–10) chosen based on previously reported work [37]. The FC-CCD design composed of factorial (3^k^), axial (2k), and replicated central (k) runs for the k number of investigated factors. The CCD “star” points outside this experimental domain and the design points at the center of the experimental domain make it possible to estimate the curvature of the response surface [33,38]. Moreover, the FC-CCD has advantages over other forms of the CCD, as its star points are at the center of each face of the factorial space (i.e., α = ± 1), it requires fewer levels of each factor (3 against 5 for other design), and also it can also be achieved by augmenting an existing factorial or resolution V design data with appropriate star points [33,38]. Table 1 provides the experimental data points required to implement the FC-CCD for the phenol uptake onto SBAC-MgAlFe-LDH, which ran in triplicate. The RSM models development using Equation (1), statistical ANOVA analysis, and optimization were undertaken using Design-Expert version 9. Equation (1):

(1) $$e=\beta_{0}+\overset{k}{\underset{i=1}{\sum }}\beta_{i}x_{i}+\overset{k}{\underset{i=1}{\sum }}\beta_{i}x_{i}^{2}+\overset{k-1}{\underset{i=1}{\sum }}\overset{k}{\underset{j=2}{\sum }}\beta_{\mathrm{ij}}x_{i}x_{j}+\varepsilon$$

where q_e_ = developed model predicted SBAC-MgAlFe-LDH adsorption capacity for phenol uptake; β_ii_, β_ij_, β_i,_ and β_0_ are the developed q_e_ model’s coefficients; x_j_, x_i_, = operational conditions. 

### 2.5. Phenol Adsorption Experiments 

Initially, phenol stock solution 1000 mg/L was prepared from which required concentrations for experimentation (20–125 mg/L) were prepared via serial dilution using Millipore machine filtered deionized water. For each test run under the conditions in Table 1, the phenol solution with a target concentration and pH set using 0.1M NaOH or 0.1M HNO_3_ was transferred to a 50 mL glass flask. The flask was firmly placed in an SK-600 Benchtop and then agitated at 120 rpm. Based on a preliminary investigation of the influence of dosage and time, a fixed dosage of 10 mg and an equilibrium time of 180 min were used for all the RSM experiments. At the end of each test run, the supernatant water was immediately separated from the spent adsorbent, first by filtering through a 0.45-micron filter, which was then followed by centrifuge separation operated for 5 min at 3500 rpm. The residual phenol concentration in each sample was determined using well-calibrated (R^2^ > 0.9998) HPLC (Thermo Scientific) UltiMate™ 3000 and a photodiode array detector set at 270 nm wavelength. 

### 2.6. Adsorption Kinetics and Equilibrium 

The phenol uptake kinetics onto the SBAC-MgAlFe-LDH was investigated using four (4) different non-linear equations that included pseudo-first and pseudo-second-order [39], intraparticle diffusion and Elovich [40], and Avrami fractional-order [41] models. Meanwhile, to further comprehend the phenol interaction with the SBAC-MgAlFe surface and elucidate the uptake mechanism, Temkin [42], Freundlich [43], Redlich and Peterson [44], Langmuir [45], and Liu [46] equilibrium models were employed.

## 3. Results and Discussion

### 3.1. Characterization of Prepared Adsorbent before and after Phenol Uptake 

**Figure 1:**
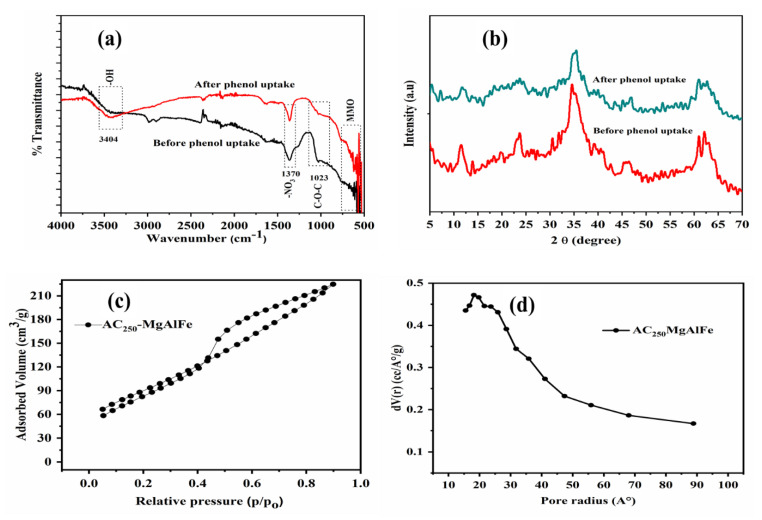
SBAC-MgAlFe characterization results: (**a**) XRD, (**b**) FTIR, (**c**) N_2_ adsorption–desorption isotherms, (**d**) pore size distribution.

The SBAC-MgAlFe-LDH clear FTIR spectrum peaks that are presented in Figure 1a indicate the abundance of surface functional groups of the ternary LDH spectrum before and after phenol uptake. The FTIR on the fresh composite exhibited peak of interlayers at 1370 cm^−1^ and 3404 cm^−1^ assigned nitrate anions (${\mathrm{NO}}_{3}^{-}$) and hydroxyl groups (OH) binding onto the metal ions of the LDH (Al, Mg, or Fe) [47]. However, for the loaded SBAC-MgAlFe-LDH that was evidently indicated by the reduced $({\mathrm{NO}}_{3}^{-}$) anions in the composite, the high and clearer intensity of the -OH groups was attributed to the existence of the higher content of the SBAC-MgAlFe-LDH in the resulting composite. Meanwhile, mixed metal oxides are indicated by the peaks located below 600 cm^−1^; the C-O-C group’s presence is attributed to the peak observed at 1023 cm^−1^ [48]. The existence of these identified surface functional groups indicates the potential of the new adsorbent as a candidate material for organic compounds uptake from water. As phenol was removed from the aqueous phase, there was an obvious transformation of the fresh SBAC-MgAlFe-LDH prior to the absorption, suggesting a possible contribution of surface functionalities for the phenol uptake [49,50]. 

The SBAC-MgAlFe-LDH diffraction peaks are displayed in Figure 1b. The peaks at 2θ values of 62.18°, 57.42°, 51.77°, 43.04°, 38.39°, 35.28°, and 30.63° are mainly attributed to the patterns of the nanoparticles [17]. The weak yet broad peak located at 11.23° corresponds to the graphitic carbon of the SBAC index plan. The XRD results suggests effective integration of the MgAlFe-LDH with the SBAC, resulting in the excellent crystalline structure of the composite. The measured BET specific surface area of 320.58 m^2^/g, pore volume 0.278 cm^3^/g, and pore radius 17.32 nm (based on BJH) for the composite SBAC-MgAlFe-LDH are a significant improvement over the values obtained for the SBAC alone as 254.3 m^2^/g, 0.14cm^3^/g, and 117.59 nm. The N_2_ isotherm trend suggests a type-IV hysteresis loop (Figure 1c), while the pore size distribution (Figure 1d) establishes that the SBAC-MgAlFe-LDH composites are characterized as mesoporous.

**Figure 2:**
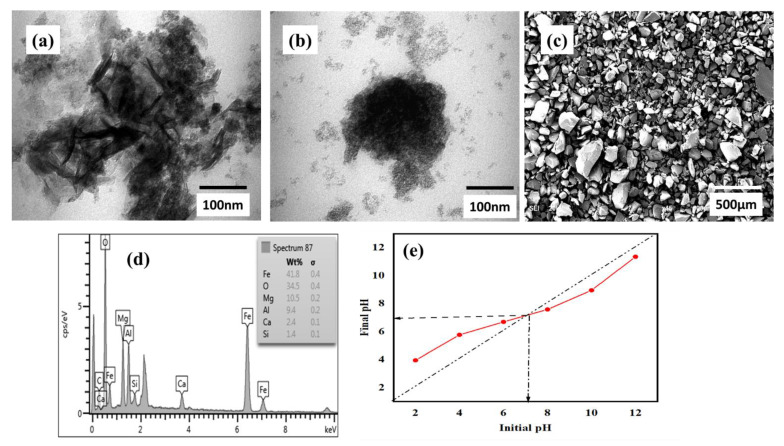
SBAC-MgAlFe: (**a**) TEM before phenol uptake, (**b**) TEM after phenol uptake, (**c**) SEM image, (**d**) EDS analyses, (**e**) Point of zero charge.

The SEM photo depicted in Figure 2c implies a heterogeneous, highly porous, and rough surface morphology, agreeing with the XRD patterns (Figure 1b). Moreover, the TEM analysis in Figure 2a suggests that the SBAC-MgAlFe-LDH nanoparticles were uniformly and homogenously dispersed and framed within the interlayers of the MgAlFe-LDH with no indication of aggregation in the resulting composite, as required for LDH composites for enhancing required adsorptive characteristics of adsorbents. The EDS chemical composition analyses (Figure 2d) provided indicated the dominance of the chemical elements, Fe, Mg, and Al, which forms the basis of the SBAC-MgAlFe-LDH. Thus, the observed improved physicochemical characteristics could be attributed to the uniform distribution of the SBAC nanoparticles into MgAlFe-LDH layers.

### 3.2. Development and Validation of RSM Model for Phenol Uptake 

**Figure 3:**
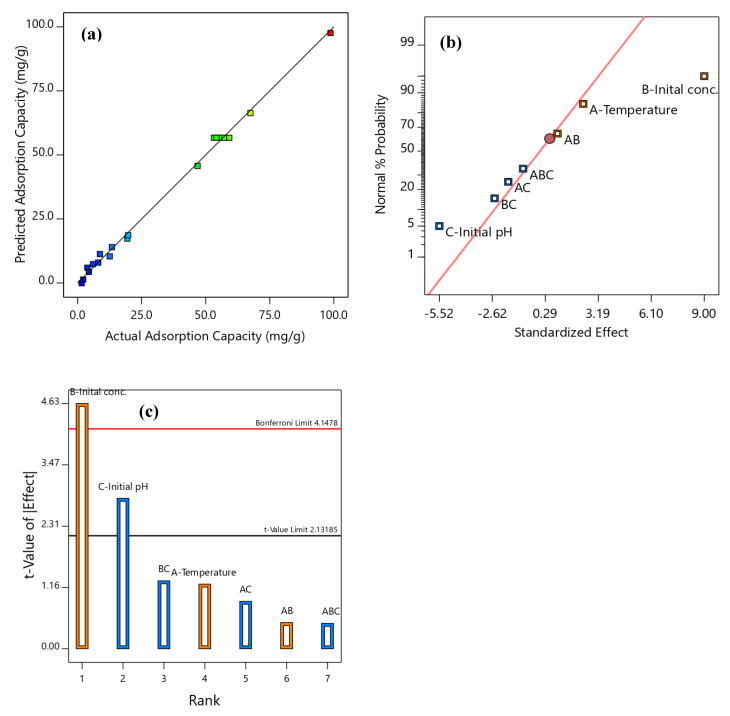
SBAC-MgAlFe-LDH adsorption capacity model’s (**a**) Experimental (actual) versus predicted values, (**b**) Probability normal plot, (**c**) Pareto chart.

The RSM regression model in Equation (1) was employed to fit in the obtained SBAC-MgAlFe-LDH phenol uptake data from which Equation (2) was obtained as the best-fitted model. The lower residuals, as provided in Table 1 and Figure 3a, indicate the closeness between the actual and model’s predicted values which suggests the high prediction ability of the developed phenol uptake RSM model. This is further corroborated via considerations of the significance of the model’s term and the insignificance of lack-of-fit (LOF); *p*-values < 0.0001 and 0.5066 were established at 5% (*p*-value < 0.05) (Table 2), respectively [34]. The analysis of variance (ANOVA) presented in Table 2 also shows that the influence of all the investigated parameters was established as all their *p*-values are <0.05 [34,37]. The developed model implies that the coded model’s terms A, B, C, C², A²B, and AB² are the primary significant model terms. Thus, the best phenol uptake model was a reduced cubic model that included only terms that significantly influence the model directly or indirectly. This means that some of the included terms, even though they are insignificant on their own, must be included as they are hierarchical terms that are indirectly reflected in higher terms that were found to significantly influence the model. In this case of the presented phenol uptake model, AB and A^2^ are insignificant; however, A²B and AB² are highly significant terms that greatly contributed to the final model quality. Accordingly, terms AB and A^2^ must be associated with the model as they contributed to the higher cubic terms that are significant; otherwise, if not included, the non-hierarchical coded model’s predictions are more unlikely to match the actual model predictions [34].

q_e_ (mg/g) = +56.70 + 10.32A + 39.48B − 3.02C + 0.48AB − 0.87AC − 1.24BC − 0.6644A² +1.44B² − 49.32C² − 34.98A²B − 9.14AB² (2)

In addition, the high predictive ability of the model is manifested in high values and closeness of the different coefficients of determination R^2^ (0.998), R^2^-predicted (0.993), and R^2^-adjusted (0.959) (as provided in Table 2). Thus, this implies that the values of both the biased and non-bias R^2^ and well the LOF are in conformity with one another, thereby meeting the requirements for RSM predictive models [51]. Meanwhile, the normal probability plot depicted in Figure 3b implies that the model satisfied the assumption of normality. On the other hand, the linear model’s normal probability plot shown in Figure 3b implies meeting the normality distribution assumption of the phenol uptake experimental data [34,52,53]. Similarly, the adequate precision of measures 51.149 (>4) fulfilled the signal-to-noise ratio requirements, and the CV = 7.56% indicates the suitability and adequacy of the employability of the model for navigating the design space. Collectively, these checks imply that the developed phenol uptake model can adequately represent the experimental data obtained for evaluation, assessments, and drawing meaningful conclusions on phenol uptake by the SBAC-MgAlFe-LDH adsorbent.

### 3.3. Influence of Operating Parameters on SBAC-MgAlFe-LDH Phenol Uptake

The influence of SBAC-MgAlFe-LDH dosage and adsorption time is depicted in Figure 2e and Figure 3a, respectively. These results (10–25 mg/L) indicated that as the dosage was increased, the adsorption capacity decreased. Meanwhile, at 10 mg dosage, increasing the time yielded higher capacity for phenol uptake, which equilibrated at 180 mg/L. As a result, the dosage of 10 mg and reaction time of 180 min were used for all RSM experiments. 

**Figure 4:**
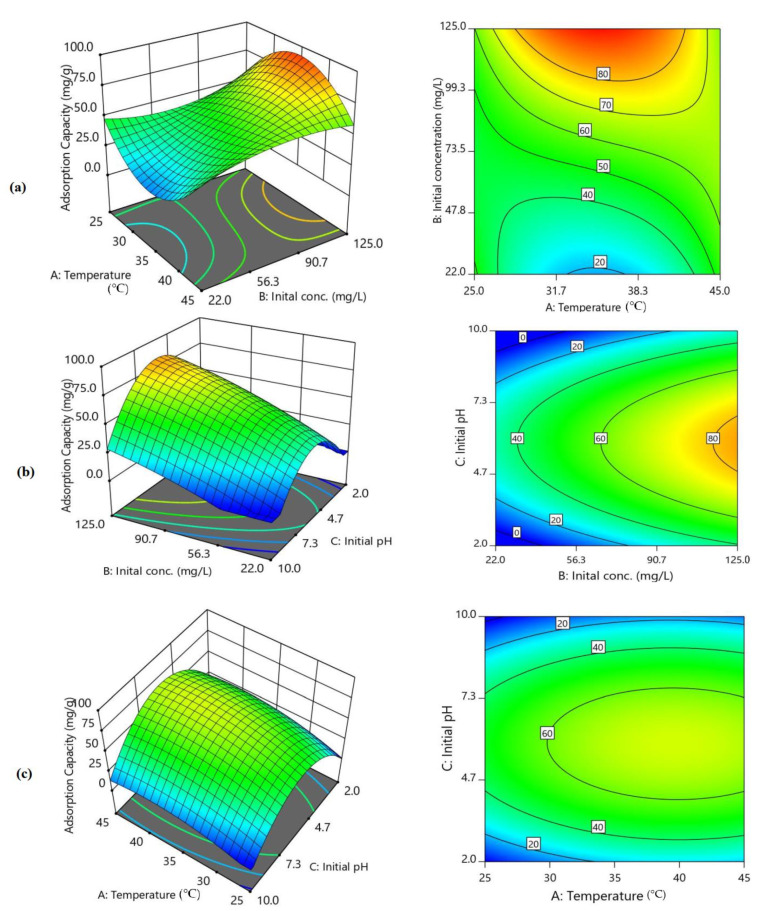
Effect of (**a**) Temperature vs. initial concentration at fixed pH 6, (**b**) Initial concentration vs. initial pH, (**c**) Temperature vs. initial pH on SBAC-MgAlFe capacity for phenol uptake.

The influences of the operational conditions (temperature, initial phenol concentration, and pH) on the performance of SBAC-MgAlFe-LDH for phenol uptake are presented in the Pareto chart (Figure 3b), and the contours and 3D plots are presented in Figure 4a–c. The Pareto chart depicts visual comparative hierarchical contributions of the single, binary and higher interactions of the RSM model’s parameters on the phenol uptake capacity. The higher the Pareto chart’s bar, the higher the relative contribution of an investigated parameter on the model’s performance, [34,52]. Hence, parameter B (initial concentration) has the strongest influence, which is followed by factor C (initial pH).

Meanwhile, the influence of the interaction effects follows the order BC > AB > AB. This shows that parameters B and C are the major influencing factors outweighing temperature (A), whose influence was less, comparatively. Moreover, the Pareto chart shows that factors A, B, and AB (orange bar) and factors C, BC, and AC (blue bars) have an antagonistic and synergetic influence on the SBAC-MgAlFe-LDH adsorption capacity, respectively, which dependently corroborates the ANOVA analyses as well as the developed model’s terms coefficients (Equation (1)). The Pareto chart t-test cut line indicated that besides the normal t-test (t = 1.367), the more conservative Bonferroni correction limit (t = 2.92) further establishes the stronger influences of initial concentration and initial pH on phenol uptake by the SBAC-MgAlFe-LDH [34,52].

The 3D curves and two-counter plots provided in Figure 4a–c depict visual influences of changing of the two parameters investigated on q_e_ while the other factor was fixed. At the fixed lowest initial pH 2 and when the initial concentration was at the lowest level, the obtainable q_e_ was the lowest (1.56–6.00 mg/g) and was not significantly affected by temperature changes (Table 2). However, at pH 6 (Figure 4a), temperature (A) and initial concentration (B) greatly influenced the q_e_ value, which resulted in the highest obtainable capacity of 98.7 mg/g at the highest phenol concentration of 125 mg/L phenol and mid-level temperature of 35 °C. Figure 4a implies that the best performances of the adsorbent at the lowest investigated phenol concentration (q_e_ = 50 mg/g) was achievable at pH 6 and 25 °C (or 45 °C), while at the mid-value temperature of 35 °C, the q_e_ drastically dwindled to the low value. Meanwhile, Figure 4b depicts the stronger influence of interaction between factors B and C, which clearly shows the direct positive direct dependency of the q_e_ on initial phenol concentration with the best performance attached at 125 mg/L phenol concentration, confirming the earlier observation. On the other hand, as the pH was raised from 2, the performance climax was achieved when it reached the value of 6 before it started decreasing and returning to the initial lower value at pH 10. Moreover, at a fixed initial phenol concentration of 73.5 mg/L, Figure 4c further reaffirms the stronger curvature influence of initial pH (the higher model’s C^2^ coefficient) as a result of interaction with the temperature at a fixed initial concentration. Figure 4c also revealed the stronger influence of initial pH compared to the temperature, which exerted less effect, and it also showed that the optimal pH was pH 6. These trends clearly corroborated the Pareto chart (Figure 3c), which suggests higher relative contributions of factors B and C on the adsorptive performance of the SBAC-MgAlFe-LDH compared to factor A. Thus, jointly, Figure 3b,c and Figure 4, and ANOVA (Table 2) show that temperature variation evidently exerted a lower influence on the LDH phenol adsorptive performance compared to initial pH and initial phenol concentrations. For the best performance of the SBAC-MgAlFe-LDH for phenol uptake, the values of factors C, B, and A should be preferably at 6, 125 mg/L, and 35 °C, respectively.

The q_e_ enhancement as a result of an increase in initial phenol concentration was ascribed to an improved induction of more contacts among phenol molecules and the active SBAC-MgAlFe-LDH sites possessing abundant functional groups as more phenol particles were introduced in the aqueous phase solution [54].

The dependencies of the SBAC-MgAlFe-LDH phenol uptake capacity changes with initial pH can be deduced based on the phenol speciation and surface charge of the SBAC-MgAlFe-LD. Thus, the results of the drift method employed to determine the point of zero charge (pH_pzc_) of the SBAC-MgAlFe-LDH suggest a pH_pzc_ = 7.09 (Figure 2e). Accordingly, this result implies that when the pH was below the neutral point, the charges on the SBAC-MgAlFe-LDH surface becomes positive due to protonation, which implies stronger electrostatic repulsion between phenol molecules and the surface of the SBAC-MgAlFe-LDH composite, which resulted in lower q_e_ [17,25]. However, at pH 6, which is closer to the pH_pzc_, a significant reduction in the positive charge on the surface of the adsorbent could be attributed to the higher performance of the SBAC-MgAlFe-LDH at the mid-value pH, as observed earlier.

Meanwhile, as the pH was increased beyond 6, the gradual transformation of the active SBAC-MgAlFe-LDH sites to negative charge might have induced repulsive electrostatic forces between phenol molecules and the LDH sites, thus leading to the observed decreased in the q_e_. Similar behavior for phenol uptake dependency on initial pH has been reported earlier [17]. The results indicated that electrostatic attraction is not associated to phenol uptake onto an SBAC-MgAlFe LDH composite. Interestingly, higher phenol uptake at pH 6 is mainly associated to a lower protonated surface, which allows phenol molecules to easily interact with composite surface functional groups (OH, MMO, and C-O-C) [20,55]. Generally, the good q_e_ at the best operational conditions was attributed to the successful intercalation of SBAC onto the interlayers of MgALFe-LDH, thereby improving its adsorptive characteristics.

### 3.4. RSM Optimization

Numerical optimization has been an indispensable tool for multivariate problems [34,56]. This is due to the intricacies associated with the identification of optimal operational points, which necessitated the simultaneous examination of all independent and dependent variables data. Consequently, optimization for SBAC-MgAlFe-LDH phenol uptake was performed under different operational conditions using the Design-Expert^®^ software numerical optimization function, which is called the “desirability function”. Thus, the desirability function capabilities were implemented under five (5) different scenarios, as presented in Table 3. According to the respective scenarios’ target goals and constraints (collectively), the well-defined and strong desirability algorithm that navigates the data finds the best solution(s) and ranks them according to the value of the “desirability” parameter that is between 1 and 0, which are designated as best to worst solutions in satisfying the optimization criteria and target goals. Accordingly, the maximum phenol uptake was set as the targeted objectives, while a variety of objectives and constraints for operating are targeted for the five (5) different scenarios 1 to 5 (Table 3). The results for the different scenarios suggest that higher initial phenol concentration and mid-value pH provide a higher desirability of the optimal solution and thus the best uptake capacity. Meanwhile, for lower initial phenol concentrations, the temperature has minimal influence on the achievable phenol uptake capacity. Thus, the highest uptake capacity obtained is inconsistent with the developed RSM model analyses presented earlier with the condition of optimality selected as A = 35 °C, B = 125 mg/L, and C = 6, which is employed for understanding mechanisms of phenol uptake onto SBAC-MgAlFe-LDH via equilibrium and kinetics studies. 

### 3.5. Adsorption Kinetics and Equilibrium Studies 

**Figure 5:**
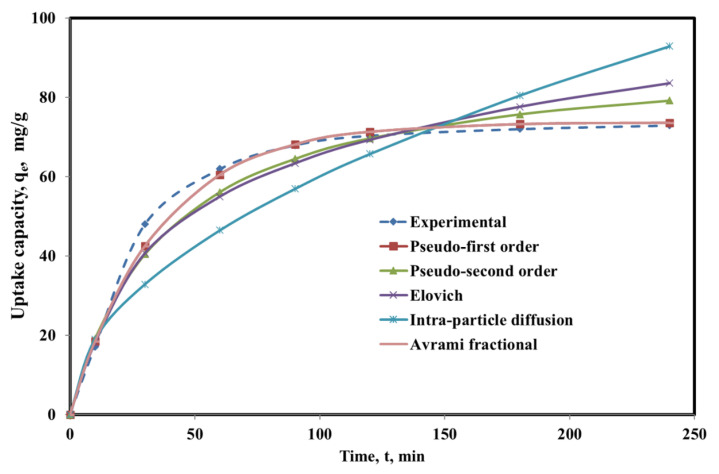
Plots for experimental data vs. predictions for non-linear kinetic models for SBAC-MgAlFe phenol uptake.

Kinetics of phenol uptake onto the SBAC-MgAlFe-LDH was studied using five (5) non-linear forms of popular kinetic models [39,40,41,57]. The kinetic models’ fittings against the experimental values of the four (4) best fitted models are displayed in Figure 5, while the respective models’ parameters are provided in Table 4. Considering the higher R^2^ = 0.98 and lower RMSE = 2.706 for the pseudo-first-order model implies that the kinetics of phenol uptake onto the SBAC-MgAlFe-LDH can be explained by the pseudo-first-order model. Interestingly, the Avrami model performance and model’s parameters match that of the first order (Table 4), reaffirming the first-order model’s better representation of the experimental data and its suitability for providing insight into the phenol uptake kinetics. 

**Figure 6:**
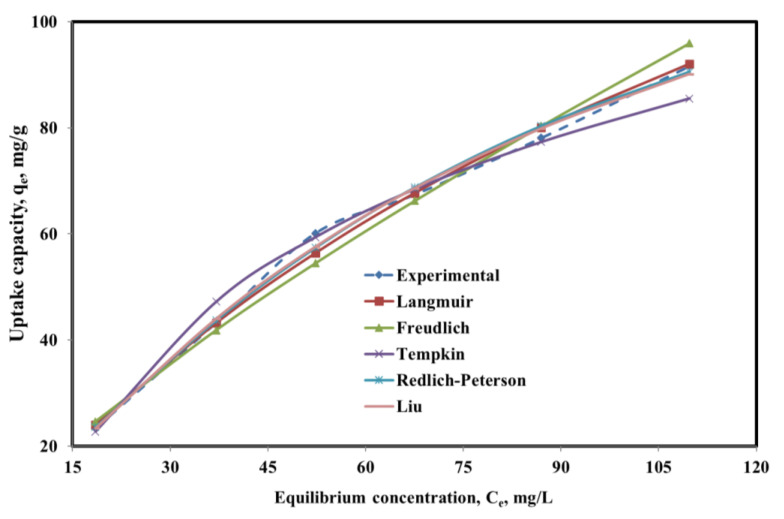
Plots for experimental data vs. predictions for non-linear equilibrium models for SBAC-MgAlFe phenol uptake.

To further elucidate the phenol uptake mechanism under equilibrium conditions and determine the maximum uptake capacity of phenol onto the SBAC-MgAlFe-LDH, non-linear forms of the five (5) different popular equilibrium models were employed in this study [35,58]. The fittings of these isotherm models are depicted in Figure 6, while their corresponding models’ parameters obtained are presented in Table 5. In terms of R^2^ and RMSE, the predictive performance of these models follows the order: Liu > Redlich–Peterson > Langmuir > Freundlich > Tempkin. The Liu and Redlich–Peterson models represent a combination of Langmuir and Freundlich models distinctly at either higher or lower concentration. This analysis implies that SBAC-MgAlFe-LDH phenol uptake can be satisfactorily explained by Liu (R^2^ = 0.996 and RMSE = 1.67) [46], Langmuir (R^2^ = 0.995 and RMSE = 1.91), and Redlich–Peterson (R^2^ = 0.995 and RMSE = 1.91) [44]. The better fittings for the Liu [46] and Redlich–Peterson [44] models are in line with earlier findings for phenolic compounds uptake by SBAC [22]. The maximum achievable monolayer phenol uptake capacity (q_max_) for SBAC-MgAlFe-LDH as per the Langmuir model was estimated at 216.76 (Table 5), even though Figure 6 indicates that the adsorption isotherms are not complete and were just reaching the maximum adsorption capacity.

A comparison of SBAC-MgAlFe-LDH phenol uptake capacity with other sewage sludge-based adsorbents produced using different activation methods is provided in Table 6. The ternary SBAC-MgAlFe-LDH reported herein possessing 216.76 mg/g capacity for phenol sorption implies an effective composite material for the effective removal of phenol from water. This is a significant improvement over SBAC reported capacities using conventional heating = 34.36 mg/g and microwave [22] and activation using CO_2_ = 32.4 mg/g [23]; chemical agents such as ZnCl_2_ = 20.95–81.6 mg/g [24,25], citric acid–ZnCl_2_ mixture [26] = 2.01 mmol/g, NaOH = 17.82–96.15 mg/g [25,27], H_2_SO_4_ = 26.16 mg/g [28], polymer flocculants = 132.33 [29], and ZnCl_2_-activated SBAC-MgFe-LDH = 138.69 mg/g [17]. Thus, the high uptake capacity exhibited by the NaOH SBAC-LDH reported in this study was ascribed to the successful synergistic influence of SBAC and the MgAlFe-based LDH composite, which yielded improved and abundant surface functional groups that supported the adsorption of more phenol molecules from water. 

### 3.6. Thermodynamics and Regeneration Studies of SBAC-MgAlFe-LDH Composite 

**Figure 7:**
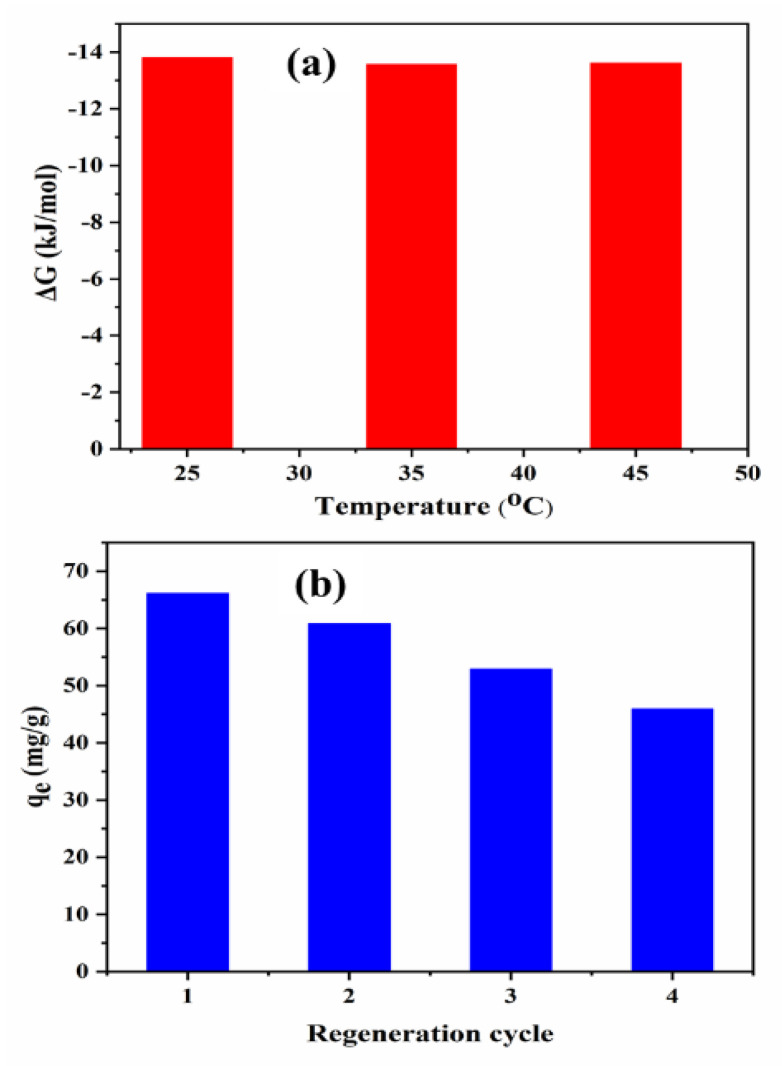
(**a**) Thermodynamic coefficients and (**b**) Consecutive regeneration and reusing of SBAC-MgAlFe for phenol uptake.

At different experiment temperatures, 25, 35, and 45 °C, and fixed other conditions as per the optimal RSM conditions of initial phenol concentration of 125 mg/L and pH 6, the thermodynamics of SBAC-MgAlFe-LDH composite phenol uptake were undertaken. At the respective different temperatures, the Gibbs free ΔG° values are close to each other with values of −5.33, −5.53 and −5.77 kJ/mol, respectively, yielding an energy enthalpy ΔH = 16.52 (kJ/mol) and entropy change ΔS = −0.105 (kJ/mol) obtained based on plotting the ΔG° values against the respective temperatures. The obtained parameters are given in Figure 7a. In general, the ΔG° value that is in the range of 0 to −20 kJ/mol corresponds to physical adsorption [59,60]. Moreover, the value of the ΔH is within the range of 2.1–20.9 kJ mol^−1^, which further establishes the physical nature of phenol uptake by SBAC-MgAlFe-LDH. All the ΔG° values are negative, indicating the spontaneous and favorable nature of the phenol uptake by the SBAC-MgAlFe-LDH composite [59,60]. Additionally, as the temperature was raised, there was an observed drop in the ΔG° values. Meanwhile, there was also positive +ΔH and absolute lower ΔG° (<20 kJ/mol), implying the endothermic nature of the phenol uptake.

The regeneration of the SBAC-MgAlFe-LDH for phenol uptake was conducted for consecutive recycles adsorption onto the SBAC-MgAlFe-LDH and subsequent desorption using ethanol (95% solution). The detailed procedure adopted to achieve this part has been reported elsewhere [17]. The result presented in Figure 7b shows that the initial phenol uptake capacity for the fresh adsorbent of 66.12 mg/g decreased to 60.81, 52.88, and 45.88 mg/g after the first, second, and third regeneration and recycling. This is about 8.03%, 20.02%, and 30.611% of the original capacity, indicating the higher reusability potential of the SBAC-MgAlFe-LDH compared with the original capacities of similar adsorbents (Table 6).

### 3.7. Possible Mechanisms of Phenol Uptake 

The synergetic effects of SBAC and the MgAlFe-based LDH resulted in improved and abundant surface functional groups that favored a high uptake of phenol molecules from the aqueous phase through multiple mechanisms involving surface adsorption and π–π interactions [20,22,55]. To further elaborate the possible adsorption mechanism of phenol, the energy of adsorption (E) was estimated from the linear form of the Dubinin–Radushkevich (DR) isotherm model (R^2^ = 0.986) and characterization of SBAC-MgAlFe-LDH before and after phenol adsorption (Figure 1 and Figure 2). The value of E at optimized adsorption conditions (pH 6, time 120 min, and temperature 35 °C) is found to be 40.82 (kJ/mol), suggesting that the possible main phenol adsorption mechanism onto SBAC-MgAlFe-LDH is involved in physisorption (π–π interactions) as a result of the phenol aromatic ring interaction with that of the SBAC-MgAlFe-LDH via charge transfer, dispersive-force, and polar attractions [17,22,29,57]. The -OH groups on the SBAC-MgAlFe-LDH surface (FTIR in Figure 1a) act as electron donors are susceptible to enhancing the aromatic-ring π-donating intensity, thereby increasing the phenol attraction onto the SBAC-MgAlFe-LDH [22,61]. Additionally, the well-established hydrophobicity of phenol provides another dimension of phenol uptake onto the SBAC-MgAlFe-LDH, as this property enhances the affinity between phenol and SBAC-MgAlFe-LDH [22,61]. Similar mechanisms for uptake phenol by sludge-based adsorbents have been postulated by other authors [22,29]. 

## 4. Conclusions

In this study, sewage sludge-based activated carbon (SBAC)-MgAlFe-LDH composite was synthesized and evaluated for the removal of phenol from the water via RSM modeling. The effect of operating conditions on phenol uptake capacity well fitted the reduced cubic model, whose temperature exerted a lower influence on the SBAC-MgAlFe-LDH phenol uptake performance compared to initial pH and initial phenol concentrations. While the pseudo-first-order and Avrami fractional models provided a better comparative representation of the phenol uptake kinetic, the Langmuir model fit the equilibrium data better. Compared with many other similar LDHs adsorbents in the literature, the obtained maximum adsorption of 216.76 mg/g indicates a significant improvement for phenol uptake by an SBAC-MgAlFe-LDH. This high uptake capacity exhibited by the SBAC-LDH was attributed to the effective synergetic effects of SBAC and the MgAlFe-based LDH, which yielded improved and abundant surface functional groups that favored the uptake of phenol molecules from the aqueous phase. These results suggest the potential of SBAC-MgAlFe-LDH as an excellent material for the effective removal of phenol water.

## Figures and Tables

**Table 1:** FC-CCD RSM experimental design matrix and results for phenol uptake onto Na-SBAC-MgAlFe-LDH.

**Factor**		**Lower Level (−1)**		**Mid-Level (0)**	**High-Lower (+1)**
A	Temperature (°C)	25		35	45
B	Initial phenol conc. (mg/L)	22.4		73.7	125
C	Initial pH	2		6	10
	**Operating Conditions**			**Responses**	
				**Adsorption capacity (mg/g)**	
**Adsorption Test**	**A**	**B**	**C**	**Predicted**	
1	35	22	6	18.65	
2	35	73.5	6	56.7	
3	35	73.5	10	4.35	
4	25	73.5	6	45.71	
5	35	73.5	6	56.7	
6	35	125	6	97.61	
7	35	73.5	6	56.7	
8	25	125	2	14.38	
9	45	22	10	1.7	
10	45	22	2	7.00	
11	45	125	2	19.45	
12	25	125	10	7.58	
13	35	73.5	2	10.4	
14	25	22	2	3.85	
15	35	73.5	6	56.7	
16	45	125	10	9.17	
17	35	73.5	6	56.7	
18	25	22	10	2.03	
19	45	73.5	6	66.35	

**Table 2:** ANOVA for a reduced cubic model for SBAC-MgAlFe-LDH phenol adsorption capacity model.

Variation Source	F-Value	*p*-Value
Model	243.92	<0.0001 ^a^
A-Temperature	38.26	0.0005 ^a^
B-Initial conc.	559.94	<0.0001 ^a^
C-Initial pH	16.43	0.0049 ^a^
AB	0.3311	0.5830 ^b^
A²	0.2166	0.6558 ^b^
B²	1.01	0.3480 ^b^
C²	1194.05	<0.0001 ^a^
A²B	351.66	<0.0001 ^a^
AB²	23.98	0.0018 ^a^
LOF	0.9226	0.5066 ^a^
R^2^ = 0.9978;	R^2^-Adjusted = 0.993;	R^2^-Predicted = 0. 959

^a^ Significance established at 5% (*p*-value < 0.05); ^b^ Significance established based on higher interaction effects (*p*-value < 0.05); LOF = Lack of fit.

**Table 3:** RSM predicted numerical optimization scenarios result for SBAC-MgAlFe-LDH phenol uptake.

Operational Parameter	Target Goals for Each Scenario					Scenario Optimization Results				
	1	2	3	4	5	1	2	3	4	5
A	In range	minimize	minimize	minimize	In range	45	25	25	28.5	35
B	22.4	22.4	73.7	125	In range	22.5	22.4	73.7	125	125
C	In range	In range	In range	In range	In range	5.89	5.963	5.91	5.848	5.82
Phenol Uptake, q_e_, mg/g	maximize	maximize	maximize	maximize	maximize	53.91	52.138	45.731	81.831	97.728
Desirability	-	-	-	-	-	0.539	0.722	0.674	0.824	0.990

**Table 4:** Parameters for different non-linear kinetic model fittings under the optimal conditions.

Model	Mathematical Representation	Parameter	Value
Pseudo-First Order	$q_{t}=(1+e^{-k_{1}t}$)	q_e_ (mg/g)	73.65
		k_1_	0.0287
		R^2^	0.980
		RMSE	2.706
Pseudo-Second Order	$q_{t}=\frac{q_{e}{}^{2}k_{2}{}^{\;}t}{1+q_{e}k_{2}{}^{\;}t}$	q_e_ (mg/g)	91.7331
		k_2_	0.0003
		R^2^	0.9401
		RMSE	5.6701
Elovich	$q_{t}=\frac{1}{\beta }\ln\left(\alpha \beta t\right)$	α	4.9400
		β	0.0485
		R^2^	0.9290
		RMSE	125.2419
Intra-Particle Diffusion	q_t_ = k_d_ t^1/2^ + C	K_d_	5.9960
		C	0.0009
		R^2^	0.7063
		RMSE	11.6561
Avrami Fractional	q_e_= q_max_ [(1 − exp(K_av_ t)]^n^	q_e_	73.65
		K_av_	0.0379
		n	0.758
		R^2^	0.980
		RSME	2.717

**Table 5:** Parameters for different non-linear equilibrium models under the optimal conditions.

Model	Mathematical Representation	Parameter	Value
Langmuir	$q_{e}=\frac{q_{\max}.K_{L}.C_{e}}{1+K_{L.}C_{e}}$	q_max, (mg/g)_	216.76
		K_L_	0.0067
		R^2^	0.9976
		RMSE	1.9100
Freundlich	$q_{e}=K_{F.}C_{e}{}^{\frac{1}{n}}$	K_F_	2.6567
		1/n	0.7635
		R^2^	0.9867
		RMSE	2.9146
Tempkin	$q_{e}=B.\ln\left(A.C_{e}\right)$	A	0.1030
		B	35.2738
		R^2^	0.9832
		RMSE	0.0041
Redlich-Peterson	$q_{e}=\frac{K_{R}.C_{e}}{1+a_{R}C_{e}^{G}}$	K_R_	1.458
		a_R_	0.0009
		G	0.989
		R^2^	0.9948
		RMSE	1.910
Liu	$q_{e}=\frac{q_{\max}.{\left(K_{g}.C_{e}\right)}^{n_{L}}}{1+{\left(K_{g}.C_{e}\right)}^{n_{L}}}$	q_max_	153.94
		K_g_	0.01227
		n_L_	1.1602
		R^2^	0.996
		RMSE	1.670

**Table 6:** Comparison of SBAC-MgAlFe-LDH phenol uptake capacity with other sewage sludge-based adsorbents produced using different activation methods.

SBAC Type/Activation Agent	Uptake Capacity (mg/g)	Operational Conditions					Reference
		Initial Conc. (mg/L)	pH	Dosage (mg)	Temp (°C)	Time (min)	
ZnCl_2_-SBAC	20.95	60	8	100	25	360	[25]
NaOH-SBAC	17.82	60	8	100	25	360	[25]
ZnCl_2_-SBAC-MgFe–LDH	138.69	100	6	10	25	180	[17]
NaOH-SBAC-MgAlFe–LDH	216.76	100	6	10	35	125	This study
Conventional furnace activated SBAC	34.36	200	-	30	25	120	[22]
Microwave-activated SBAC	32.96	200	-	30	25	120	[22]
CO_2_-activated sludge	32.4	250	5	15	20	72h	[23]
H_2_SO_4_ SBAC	26.16	200	-	5g/L	20	120	[28]
Citric acid–ZnCl_2_-SBAC	189.16	0.5 to 50 mmol	4	1 g /L	30	6 h	[26]
NaOH-SBAC	96.15	-	-	10	25	60	[27]
ZnCl_2_-SBAC	81.6	2000	-	1 g/100 mL	25	48 h	[24]
Polymer flocculants activated SBAC	132.33	75	5	−	40	−	[29]

## Data Availability

Not applicable.

## Sample Availability

Samples of the compounds are available from the authors.
